# Critical Evaluation of CrAssphage as a Molecular Marker for Human-Derived Wastewater Contamination in the Aquatic Environment

**DOI:** 10.1007/s12560-019-09369-1

**Published:** 2019-02-13

**Authors:** Kata Farkas, Evelien M. Adriaenssens, David I. Walker, James E. McDonald, Shelagh K. Malham, Davey L. Jones

**Affiliations:** 1 School of Natural Sciences, Bangor University, Deiniol Road, Bangor, Gwynedd UK; 2 Microbiology Research Group, Institute of Integrative Biology, University of Liverpool, Liverpool, UK; 3 Centre for Environment, Fisheries and Aquaculture Science, Weymouth, Dorset UK; 4 School of Ocean Sciences, Bangor University, Menai Bridge, Anglesey UK; 5 UWA School of Agriculture and Environment, University of Western Australia, Crawley, Australia; 6 Present Address: Quadram Institute Bioscience, Norwich Research Park, Norwich, UK

**Keywords:** Wastewater pollution, CrAssphage, Enteric viruses, Shellfish hygiene, qPCR

## Abstract

The discharge of human-derived wastewater represents a major threat to water quality with the potential for waterborne disease outbreaks mainly associated with enteric viruses. To prevent illnesses, indicators associated with fecal contamination are monitored in polluted areas, however, their prevalence often does not correlate well with viral pathogens. In this study, we used crAssphage, a recently discovered human-specific gut-associated bacteriophage, for the surveillance of wastewater-derived viral contamination. Untreated and treated wastewater, surface water, sediment and mussel samples were collected monthly over 1 year from the Conwy River and estuary (UK) and were analyzed for crAssphage marker by quantitative PCR. This is the first long-term catchment-to-coast scale study of environmental crAssphage concentrations. CrAssphage was detected in all sample types and showed no distinct seasonal pattern. CrAssphage concentrations were 2 × 10^5^–10^9^ genome copies (gc)/L in all untreated wastewater influent and 10^7^–10^8^ gc/L in secondary treated effluent samples, 3 × 10^3^ gc/L–3 × 10^7^ gc/L in surface water samples (94% positive) and 2 × 10^2^–10^4^ gc/g sediment (68% positive) and mussel digestive tissue (79% positive). CrAssphage concentrations were 1–5 log_10_ higher than human enteric virus titers (norovirus, sapovirus, adenovirus, polyomavirus). Our results indicate that crAssphage is well suited to tracking human wastewater contamination and pollution risk assessment in aquatic environments.

**Electronic supplementary material:**

The online version of this article (10.1007/s12560-019-09369-1) contains supplementary material, which is available to authorized users.

## Introduction

Enteric viruses are the most common etiologic agents of gastroenteritis globally. They are discharged with treated and untreated wastewater into the aquatic environment where they are able to persist for long periods of time (Kotwal and Cannon 2014). Hence, these viruses are often responsible for waterborne and foodborne illnesses due to the use of contaminated recreational water, and the consumption of polluted water and shellfish (Radin 2014; Rodríguez-Lázaro et al. 2012). Over 150 human pathogenic viruses, including noroviruses (NoV), sapoviruses (SaV), rotaviruses, hepatitis A/E viruses, adenoviruses (AdV), enteroviruses and polyomaviruses, have been identified in watercourses (Rodríguez-Lázaro et al. 2012; Tran et al. 2015). As the detection and surveillance of all pathogenic viral strains is not feasible, indicators are often used for tracking wastewater contamination in the environment (Symonds and Breitbart 2015).

The crAssphage *sensu stricto* genome (RefSeq accession number NC_024711), hereafter referred to as crAssphage, was first assembled from human fecal microbiomes within the last 5 years (Dutilh et al. 2014). Further analysis of human metagenomes showed that crAssphage is a highly abundant virus in the human gut, especially in individuals living in industrialized areas (Honap et al. 2018; Stachler and Bibby 2014). Data mining of virome datasets subsequently revealed that there is a diverse group of crAss-related phages present in the human gut, potentially representing a new family of viruses (Yutin et al. 2017). The first isolated representative of this family has just been discovered, phiCrAss001, infecting the human gut bacterium *Bacteroides intestinalis* (Shkoporov et al. 2018). Following the discovery of the prevalence of crAss-like phages in the human gut, their usefulness as a tool for source tracking of human fecal pollution was recognized (Stachler and Bibby 2014). Quantitative PCR (qPCR) assays have been developed for the quantification of the crAssphage in stool samples (Cinek et al. 2018; Liang et al. 2018; Stachler et al. 2017). Assay specificity showed great variation, suggesting that some regions of the crAssphage genome may be similar to other bacteriophage genome segments, whereas other regions are indeed human-specific (Stachler et al. 2018). CrAssphage prevalence has been investigated in the aquatic environment and found at high concentrations in untreated wastewater, wastewater-contaminated stream and in stormwater (Ahmed et al. 2018; García-Aljaro et al. 2017; Stachler et al. 2018). The concentration correlated with rainfall frequency in a polluted stream in a 30-day monitoring trial (Stachler et al. 2018). Nonetheless, crAssphage has not been used for long-term monitoring of wastewater-contaminated environments.

The aim of the research described here was to assess the usefulness of a crAssphage marker specifically as an indicator for wastewater-associated viral contamination in the aquatic environment. CrAssphage marker concentrations were monitored in treated and untreated wastewater, riverine and estuarine water, and sediment and shellfish samples collected monthly over 1 year along the Conwy River and estuary, UK. CrAssphage marker concentrations were compared with common enteric viral contaminants, namely NoV genogroups GI and GII (NoVGI and NoVGII), SaV genogroup GI, AdV and JC polyomavirus (JCV).

## Materials and Methods

### Sample Types and Process

We investigated the presence and concentration of crAssphage in nucleic acid extracted from concentrated wastewater, surface water, sediment and shellfish (blue mussel, *Mytilus edulis*) samples that were known to be contaminated with enteric viruses. The sampling schedule, locations and sample processing are detailed in Farkas et al (2018a) and summarized in Table 1. Thirteen samples were taken of each sample type once per month over a 1-year cycle (August 2016–August 2017).

**Table 1:** Percentile of virus-positive samples in wastewater (WW), surface water (SW), sediment (Sed) and mussel (SF) samples collected in the Conwy catchment and estuary between August 2016 and August 2017

Sample code	Site	Sample type	NoVGI (%)	NoVGII (%)	SaV (%)	AdV (%)	JCV (%)	CrAssphage (%)
GI	Ganol	WW influent	62	69	38	92	100	100
BI	Betws-y-Coed	WW influent	31	62	15	85	85	100^a^
BE	Betws-y-Coed	WW effluent (AC)	31	38	0	77	85	100^a^
LI	Llanrwst	WW influent	15	54	15	85	85	100^a^
LE	Llanrwst	WW effluent (BF)	31	62	15	92	100	100
TI	Tal-y-Bont	WW influent	31	62	38	100	92	100^a^
TE	Tal-y-Bont	WW effluent (AC)	45	46	15	92	85	100^a^
SW1	Betws-y-Coed–Llugwy River	River water	15	23	8	85	54	92
SW2	Betws-y-Coed–Conwy River	River water	0	8	8	85	54	92^a^
SW3	Llanrwst	Estuarine water	15	8	0	92	92	92
SW4	Conwy	Estuarine water	8	15	0	92	62	92
Sed1	Deganwy beach	Estuarine sediment	31	15	0	77	46	62
Sed2	Morfa beach	Estuarine sediment	8	0	0	85	46	75^a^
Sed4	Conwy	Estuarine sediment	15	8	0	85	23	62
SF1	Deganwy beach	Mussel	15	15	0	85	31	77
SF2	Morfa beach	Mussel	23	8	0	85	38	83^a^

*AC* activated sludge treatment, *BF* biofilter; *n* = 13, ^a^*n* = 12. *NoV GI and GII* Norovirus GI and GII, *SaV* sapovirus, *AdV* adenovirus and *JCV* JC polyomavirus data were adopted from Farkas et al. (2018a)

The water and wastewater samples, 10 L and 1 L, respectively, were concentrated using tangential flow ultrafiltration followed by beef extract elution and polyethylene glycol (PEG) precipitation (Farkas et al. 2018). Viruses were eluted from 10 g of sediment using beef extract solution and precipitated with PEG (Farkas et al. 2017). The mussel digestive tissue samples, 2 g each, were processed as described in the ISO-TS 15216 standard (International Organizatoin for Standardization 2013). Nucleic acids were extracted from the concentrates using the MiniMag NucliSens Nucleic Acid Extraction System (BioMérieux SA, France). Assay efficiency was assessed using mengovirus as a process control, which gave > 10% recovery values (Farkas et al. 2018).

### CrAssphage Quantification

CrAssphage DNA was quantified using the CPQ_056 TaqMan primers and probe, which have shown great accuracy and little cross-reaction with animal litter (Ahmed et al. 2018; Stachler et al. 2017). The qPCR assays were carried out in a QuantStudio^®^ Flex 6 Real-Time PCR System (Applied Biosystems, USA). The 20 µL reaction mix contained 1x KAPA Probe Force qPCR mix (KAPA Biosystems, USA) with 10 pmol of the forward, 10 pmol of the reverse primers, 5 pmol of the probe, 1 µg bovine serum albumin, 50 nM ROX reference dye. The sample volume was 2 µL for wastewater influent, 4 µL for wastewater effluent and surface water, and 8 µL for sediment and mussel samples in each reaction. For quantification, dilution series of a plasmid DNA carrying the target sequence were used. Non-template controls (molecular-grade water) were added to each reaction plate. Amplification was carried out using the following thermal cycling conditions: 98 °C for 5 min, then 40 cycles of 95 °C for 15 s, 60 °C for 1 min. The assay efficiency was 90–110%.

### Data Analysis

The viral concentrations were expressed as gc/L of wastewater and surface water or gc/g of sediment or mussel digestive tissue. Linear regressions and Spearman’s rank correlation coefficients (*r*) were calculated between viral concentrations using two-tailed 95% confidence intervals in SigmaPlot 13.0 (Systat Software Inc., US).

## Results and Discussion

### CrAssphage qPCR

The main goal of this study was to evaluate the usefulness of a novel human-associated phage, the crAssphage, as a viral indicator for wastewater contamination. We, therefore, analyzed crAssphage concentrations in wastewater influent and effluent samples released into the river, and in river and estuarine water, sediment and mussel samples contaminated with wastewater, and correlated the concentrations of the crAssphage with enteric viral titres in the samples using the qPCR results. In our study, the non-template controls were negative in each assay suggesting no cross-contamination. The LOD was 2 gc/reaction and samples below that concentration were considered negative. The LOQ was 20 gc/reaction. Dilution of samples did not affect qPCR results indicating no inhibition in the wastewater effluent, sediment or mussel matrices. We utilized a well-established primer and probe set that has previously been used for the quantification of crAssphage in stool, wastewater, storm drain outfall and surface water (Ahmed et al. 2018; Stachler et al. 2017, 2018). The qPCR assay has previously shown good human specificity, with little cross-reaction with dog, gull and poultry litter (Ahmed et al. 2018; Stachler et al. 2017). Overall, the qPCR assay used for crAssphage quantification appeared to be accurate and sensitive and hence suitable for the analysis of environmental samples.

### CrAssphage Concentrations in Wastewater and in the Aquatic Environment

All wastewater influent and effluent samples were positive for crAssphage with no seasonal patterns discernible (Table 1; Table S1). In the wastewater influent samples, crAssphage concentrations varied between 2.2 × 10^5^ gc/L and 1.2 × 10^9^ gc/L, with the lowest concentrations observed in the samples collected at Llanrwst (serving approx. 4000 inhabitants) and the highest concentrations in samples derived from the Ganol WWTP (serving approx. 82,000 inhabitants). These concentrations are lower than previously observed in untreated wastewater in Tampa, Florida (10^9^–10^10^ gc/L) (Ahmed et al. 2018) and in Catalonia, Spain (10^8^–10^9^ gc/L) (García-Aljaro et al. 2017). The differences in crAssphage concentrations in untreated wastewater may be due to the differences in geographic viral distribution (Stachler and Bibby 2014), WWTP sizes and the level of urbanization, which has been shown to affect crAssphage abundance among inhabitants (Honap et al. 2018).

CrAssphage concentrations in wastewater effluent samples ranged between 1.7 × 10^5^ and 2.0 × 10^8^ gc/L. In general, we observed a very low (less than 1-log_10_) reduction of crAssphage during wastewater treatment at Llanrwst (using biofilter treatment), whereas the concentrations were reduced by up to 2-log_10_ at the Betws-y-Coed and Tal-y-Bont WWTPs (using activated sludge treatment). This suggests that there is a difference in the performance of the different types of wastewater treatment methods. To date, no quantitative studies have assessed the prevalence of crAssphage in treated wastewater.

CrAssphage was detected in 92% of all river and estuarine water samples (Table 1; Table S1). The concentration of crAssphage was approx. 1–3 log_10_ lower in the surface water relative to the treated wastewater and varied between 3.0 × 10^3^ gc/L and 2.5 × 10^7^ gc/L. The highest concentrations were observed in the SW3 samples (3.0 × 10^6^ gc/L on average) derived approximately 200 m downstream of the Llanrwst wastewater discharge point. These concentrations are consistent with the previously reported crAssphage concentrations in wastewater-polluted surface water (10^4^–10^6^ gc/L) (Stachler et al. 2018). No seasonal patterns in the crAssphage concentrations in the surface water samples were observed.

The sediment and mussel samples had more negatives than the wastewater and surface water samples (Table 1 and S1) with concentrations up to 1.9 × 10^4^ gc/g sediment or mussel digestive tissue. No seasonality or significant differences were observed among the sediment and mussel sample crAssphage concentrations. Detection of crAssphage in sediment or in mussel samples has not previously been reported.

### Comparison of Enteric Virus and CrAssphage Concentrations in Different Sample Types

**Fig. 1:**
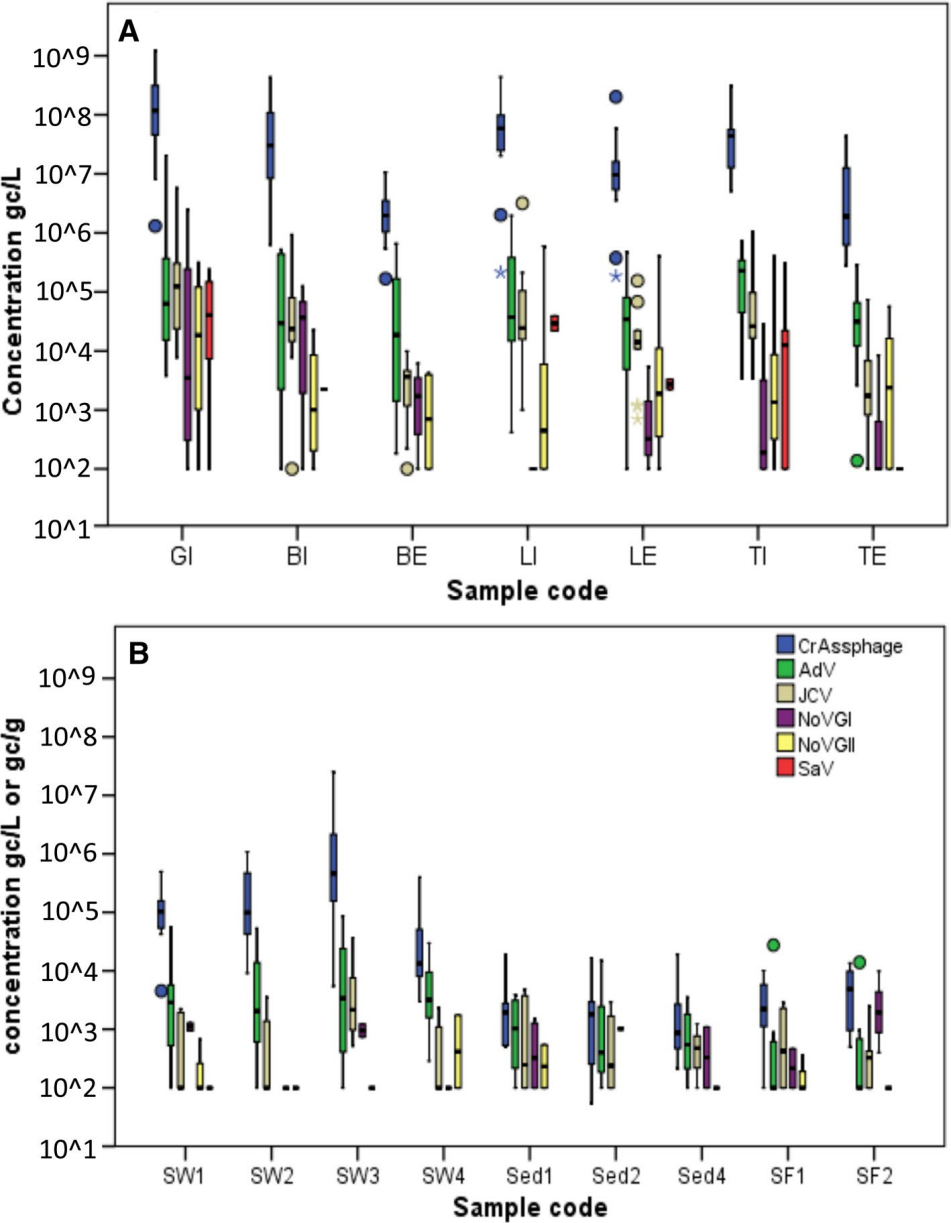
Box plot showing the median concentration (min–max) of crAssphage (blue), human adenovirus (AdV; green), JC polyomavirus (JCV; gray) norovirus GI (NoVGI; purple) and GII (NoVGII, yellow) and sapovirus (SaV; red) determined in (A) wastewater influent (GI, BI, LI and TI) and effluent (BE, LE, TE) and in (B) surface water (SW1-4), sediment (Sed1, 2, 4) and mussel (SF1, 2). Sample concentration below the limit of quantification was considered 100 gc/l or gc/g. Circles: outliers, starts extreme outliers

CrAssphage concentrations in wastewater, surface water, sediment and mussel samples were compared with the human-specific NoVGI, NoVGII, SaV, AdV and JCV concentrations of the same samples, which were determined in a previous study (Farkas et al. 2018). The enteric viruses were less prevalent and were present at lower concentrations than crAssphage. The virus-positive wastewater samples, both influent and effluent wastewater samples, had approx. 2 log_10_ higher crAssphage than enteric virus titers (Fig. 1). A weak correlation (*r* < 0.5) among viral titers was observed in wastewater influent except for the crAssphage/JCV and the AdV/SaV pairs, which showed moderate correlation (*r* = 0.50 and 0.55; Table 2 and S2). The reduction rate of all tested viruses in the WWTPs were very similar, resulting in approximately 1 log_10_ reduction during biofilter treatment and 1–2 log_10_ reduction during activated sludge treatment. Similar reduction trends have been reported in other studies on enteric virus and coliphage titers in activated sludge and biofilter-treated wastewater (Campos et al. 2016; Hewitt et al. 2011; Kitajima et al. 2014; Sidhu et al. 2017). In wastewater effluent, strong correlation was noted for crAssphage/JCV (*r* = 0.67; Table 2). The crAssphage and JCV showed a linear correlation in the untreated wastewater samples, however, such correlation was only observed in the wastewater effluents that underwent biofilter treatment (*R*^2^ = 0.81; Figure S1).

**Table 2:** Spearman correlation between crAssphage and human enteric virus titers

Sample type	Viruses	*R*	*p*
Wastewater influent	CrAssphage–AdV	0.31*	0.03
	CrAssphage–JCV	0.50***	< 0.01
	CrAssphage–NoVGI	0.26	0.07
	CrAssphage–NoVGII	0.34*	0.02
	CrAssphage–SaV	0.265	0.07
Wastewater effluent	CrAssphage–AdV	0.13	0.45
	CrAssphage–JCV	0.67***	< 0.01
	CrAssphage–NoVGI	0.20	0.23
	CrAssphage–NoVGII	0.43**	< 0.01
	CrAssphage–SaV	0.21	0.21
Surface water	CrAssphage–AdV	− 0.02	0.88
	CrAssphage–JCV	0.49***	< 0.01
	CrAssphage–NoVGI	0.08	0.55
	CrAssphage–NoVGII	0.07	0.64
	CrAssphage–SaV	− 0.07	0.63
Sediment	CrAssphage–AdV	0.37*	0.02
	CrAssphage–JCV	0.09	0.61
	CrAssphage–NoVGI	− 0.08	0.63
	CrAssphage–NoVGII	0.24	0.14

*R*: correlation coefficient. Further data are shown in Supplementary Table 2. *p < 0.05; **p < 0.01; ***p < 0.001. No SaV was detected in the sediment

In the surface water samples, crAssphage was more prevalent than the enteric viruses. Only AdV was detected in a similar number of samples to crAssphage (85–92%), whereas the detection rates of the JCV (54–92%) and the NoVs and SaV (0–23%) were lower (Table 1). The concentration of crAssphage was 1–2 log_10_ higher than the concentration of the AdV and JCV, and 2–5 log_10_ higher than that observed for NoVs and SaV (Fig. 1). In the sediment and mussel samples, the detection rate of crAssphage (62–75%) was slightly lower than the detection rate of AdV (77–85%) but higher than observed for the JCV (23–46%) and NoVs (0–31%; Table 1). The concentration of crAssphage in sediment was similar to the concentration of AdV and JCV in sediment and slightly higher in mussel (Fig. 1). Weak or no correlation was observed among viruses in surface water, sediment and shellfish samples (Table S2). A linear correlation between crAssphage and JCV was only observed in the highly polluted surface water sample (SW3), where the highest viral titers were observed (Figure S1). The lack of correlation between viral titers is probably due to the low copy numbers and the low number of positive samples.

### Environmental Implications and Future Research

In this study, the concentration of crAssphage in wastewater and in wastewater-contaminated environments was tracked over a period of 1 year for the first time. Similar to AdV and JCV, which are considered markers for human wastewater pollution (Rachmadi et al. 2016; Rames et al. 2016), crAssphage incidence displayed no seasonal patterns and was prevalent in all sample types. These findings suggest that the use of crAssphage as a fecal contamination indicator enables the assessment of pollutant transport. This was the first time crAssphage detection was addressed in sediment and in bivalve shellfish. CrAssphage was frequently detected in mussel and sediment, collected at the wastewater-contaminated areas, demonstrating that crAssphage may be a suitable fecal pollution indicator in those matrices.

Future research should explore the usefulness of qPCR assays targeting different regions of the crAssphage genome to enhance specificity, explore human specificity and exploit the recently discovered new crAss-like genomes to identify geographic differences in diversity and assess correlations between the concentrations of crAss-like phages and a wide range of enteric viruses and other viral and bacterial indicators. The prevalence of crAssphage in waters not affected by wastewater contamination also needs to be established. Furthermore, with the very recent discovery of a crAss-like phage isolate (Guerin et al. 2018) and the successful in vitro culturing of that strain (Shkoporov et al. 2018) future approaches should be focused on comparative description of crAssphage, F-RNA phage and AdV/JCV infectivity, survival and persistence in wastewater and in the aquatic environment, including surface and groundwater, and in bivalve shellfish and fresh produce. Once established, crAssphage may be a suitable marker for routine bivalve shellfish testing in commercial fisheries.

## Electronic supplementary material

Below is the link to the electronic supplementary material.

Supplementary material 1 (DOCX 61 KB)

## References

[CR1] Ahmed W, Lobos A, Senkbeil J, Peraud J, Gallard J, Harwood VJ (2018). Evaluation of the novel crAssphage marker for sewage pollution tracking in storm drain outfalls in Tampa, Florida. Water Research.

[CR2] Ahmed W, Payyappat S, Cassidy M, Besley C, Power K (2018). Novel crAssphage marker genes ascertain sewage pollution in a recreational lake receiving urban stormwater runoff. Water Research.

[CR3] Ahmed W, Zhang Q, Lobos A, Senkbeil J, Sadowsky MJ, Harwood VJ (2018). Precipitation influences pathogenic bacteria and antibiotic resistance gene abundance in storm drain outfalls in coastal sub-tropical waters. Environment International.

[CR4] Campos CJA, Avant J, Lowther J, Till D, Lees DN (2016). Human norovirus in untreated sewage and effluents from primary, secondary and tertiary treatment processes. Water Research.

[CR5] Cinek O, Mazankova K, Kramna L, Odeh R, Alassaf A, Ibekwe MAU (2018). Quantitative CrAssphage real-time PCR assay derived from data of multiple geographically distant populations. Journal of Medical Virology.

[CR6] Dutilh BE, Cassman N, McNair K, Sanchez SE, Silva GGZ, Boling L (2014). A highly abundant bacteriophage discovered in the unknown sequences of human faecal metagenomes. Nature Communications.

[CR7] Farkas K, Cooper DM, McDonald JE, Malham SK, de Rougemont A, Jones DL (2018). Seasonal and spatial dynamics of enteric viruses in wastewater and in riverine and estuarine receiving waters. Science of the Total Environment.

[CR8] Farkas K, Hassard F, McDonald JE, Malham SK, Jones DL (2017). Evaluation of molecular methods for the detection and quantification of pathogen-derived nucleic acids in sediment. Frontiers in Microbiology.

[CR9] Farkas K, Mcdonald JE, Malham SK, Jones DL (2018). Two-step concentration of complex water samples for the detection of viruses. Methods and Protocols.

[CR10] García-Aljaro C, Ballesté E, Muniesa M, Jofre J (2017). Determination of crAssphage in water samples and applicability for tracking human faecal pollution. Microbial Biotechnology.

[CR11] Guerin E, Shkoporov A, Stockdale SR, Clooney AG, Ryan J, Draper LA (2018). Biology and taxonomy of crAss-like bacteriophages, the most abundant virus in the human gut. bioRxiv.

[CR12] Hewitt J, Leonard M, Greening GE, Lewis GD (2011). Influence of wastewater treatment process and the population size on human virus profiles in wastewater. Water Research.

[CR13] Honap TP, Sankaranarayanan K, Schnorr SL, Ozga AT, Warinner C, Lewis CM (2018). Biogeographic study of human gut associated crAssphage suggests impacts from industrialization and recent expansion. bioRxiv.

[CR14] International Organizatoin for Standardization. (2013). International Organization for Standardization ISO/TS 15216-2:2013-Microbiology of food and animal feed—Horizontal method for determination of hepatitis A virus and norovirus in food using real-time RT-PCR—Part 2: Method for qualitative detection.

[CR15] Kitajima M, Iker BC, Pepper IL, Gerba CP (2014). Relative abundance and treatment reduction of viruses during wastewater treatment processes—Identification of potential viral indicators. Science of the Total Environment.

[CR16] Kotwal G, Cannon JL (2014). Environmental persistence and transfer of enteric viruses. Current Opinion in Virology.

[CR17] Liang Y, Jin X, Huang Y, Chen S (2018). Development and application of a real-time polymerase chain reaction assay for detection of a novel gut bacteriophage (crAssphage). Journal of Medical Virology.

[CR18] Rachmadi AT, Torrey JR, Kitajima M (2016). Human polyomavirus: Advantages and limitations as a human-specific viral marker in aquatic environments. Water Research.

[CR19] Radin D (2014). New trends in food-and waterborne viral outbreaks. Archives of Biological Sciences.

[CR20] Rames E, Roiko A, Stratton H, Macdonald J (2016). Technical aspects of using human adenovirus as a viral water quality indicator. Water Research.

[CR21] Rodríguez-Lázaro D, Cook N, Ruggeri FM, Sellwood J, Nasser A, Nascimento MSJ (2012). Virus hazards from food, water and other contaminated environments. FEMS Microbiology Reviews.

[CR22] Shkoporov AN, Khokhlova EV, Fitzgerald CB, Stockdale SR, Draper LA, Ross RP, Hill C (2018). ΦCrAss001 represents the most abundant bacteriophage family in the human gut and infects Bacteroides intestinalis. Nature Communications.

[CR23] Sidhu JPS, Ahmed W, Palmer A, Smith K, Hodgers L, Toze S (2017). Optimization of sampling strategy to determine pathogen removal efficacy of activated sludge treatment plant. Environmental Science and Pollution Research.

[CR24] Sidhu JPS, Sena K, Hodgers L, Palmer A, Toze S (2017). Comparative enteric viruses and coliphage removal during wastewater treatment processes in a sub-tropical environment. Science of the Total Environment.

[CR25] Stachler E, Akyon B, De Carvalho A, Ference N, Bibby K (2018). Correlation of crAssphage-based qPCR markers with culturable and molecular indicators of human fecal pollution in an impacted urban watershed. Environmental Science and Technology.

[CR26] Stachler E, Bibby K (2014). Metagenomic evaluation of the highly abundant human gut bacteriophage CrAssphage for source tracking of human fecal pollution. Environmental Science & Technology Letters.

[CR27] Stachler E, Kelty C, Sivaganesan M, Li X, Bibby K, Shanks OC (2017). Quantitative CrAssphage PCR assays for human fecal pollution measurement. Environmental Science and Technology.

[CR28] Symonds EM, Breitbart M (2015). Affordable enteric virus detection techniques are needed to support changing paradigms in water quality management. Clean Soil Air Water.

[CR29] Tran NH, Gin KYH, Ngo HH (2015). Fecal pollution source tracking toolbox for identification, evaluation and characterization of fecal contamination in receiving urban surface waters and groundwater. Science of the Total Environment.

[CR30] Yutin, N., Makarova, K. S., Gussow, A. B., Krupovic, M., & Segall, A. (2017). Discovery of an expansive family of bacteriophages that includes the most abundant viruses from the human gut. *Nature Microbiology*, 1–9. 10.1038/s41564-017-0053-y. 10.1038/s41564-017-0053-y PMC5736458 29133882

