# Supplementary material for: Critical Evaluation of CrAssphage as a Molecular Marker for Human-Derived Wastewater Contamination in the Aquatic Environment
Source: Food Environ Virol. 2019 Feb 13;11(2):113–9. doi: 10.1007/s12560-019-09369-1 (PMC6513805; doi:10.1007/s12560-019-09369-1)
Supplement: Supplementary file 1 — Supplementary material 1 (DOCX 61 KB) [file 12560_2019_9369_MOESM1_ESM.docx]

Table S1. Observed crAssphage concentrations in wastewater, surface water, sediment and shellfish samples in genome copies/litre (gc/L) or gc/g. Detected: below limit of quantification (20 gc/qPCR reaction). Negative: below the limit of detection (2 gc/qPCR reaction). SD: standard deviation. Purple: 1x10^9^ gc/L; brown: 1-5x10^8^ gc/L; red: 1-9.9x10^7^ gc/L; yellow: 1-9.9x10^6^ gc/L; green: 1-9.9x10^5^ gc/L; blue: 1-9.9x10^4^ gc/L or gc/g; pink: 1-9.9x10^3^ gc/L or gc/g; grey: 1-9.9x10^2^ gc/g.

|  | Wastewater influent (gc/L) | | | | Wastewater effluent (gc/L) | | | River water (gc/L) | | Estuarine water (gc/L) | |
| --- | --- | --- | --- | --- | --- | --- | --- | --- | --- | --- | --- |
|  | GI | BI | LI | TI | BE | LE | TE | SW1 | SW2 | SW3 | SW4 |
| Aug-16 | 8.07x10^6^ | 1.85x10^7^ | 2.15x10^5^ | 5.08x10^6^ | 3.08x10^6^ | 3.76x10^5^ | 5.54x10^5^ | 4.53E x10^3^ | 1.13x10^5^ | 3.77x10^5^ | 6.99x10^4^ |
| Sep-16 | 1.31x10^6^ | 2.72x10^6^ | 2.01x10^6^ | 1.14x10^7^ | 1.68x10^5^ | 1.85x10^5^ | 2.76x10^5^ | 1.17x10^5^ | 9.12x10^3^ | 2.52x10^5^ | 3.42x10^3^ |
| Oct-16 | 2.45x10^7^ | 1.36x10^7^ | 3.05x10^7^ | 2.53x10^7^ | 8.17x10^5^ | 1.42x10^7^ | 4.15x10^7^ | 4.24x10^4^ | 9.90x10^4^ | 1.62x10^5^ | 8.23x10^3^ |
| Nov-16 | 3.49x10^8^ | 1.01x10^7^ | not tested | 5.06x10^7^ | 7.68x10^6^ | 1.15x10^7^ | 3.14x10^7^ | 4.23x10^4^ | 2.04x10^4^ | 1.49x10^5^ | 1.65x10^4^ |
| Dec-16 | 1.18x10^8^ | not tested | 5.31x10^7^ | not tested | not tested | 8.93x10^6^ | not tested | 1.33x10^5^ | 1.08x10^6^ | 5.48x10^3^ | 3.67x10^3^ |
| Jan-17 | 3.12x10^8^ | 1.90x10^8^ | 1.43x10^8^ | 4.49x10^7^ | 4.00x10^6^ | 1.61x10^7^ | 4.38x10^7^ | negative | 4.61x10^5^ | 1.20x10^5^ | 5.12x10^4^ |
| Feb-17 | 1.40x10^8^ | 2.90x10^8^ | 8.32x10^7^ | 4.31x10^7^ | 1.82x10^6^ | 9.60x10^6^ | 1.27x10^6^ | 8.54x10^4^ | 3.43x10^4^ | 5.68x10^5^ | 2.86x10^4^ |
| Mar-17 | 1.05x10^8^ | 4.90x10^7^ | 4.20x10^7^ | 1.00x10^7^ | 1.06x10^7^ | 5.89x10^7^ | 4.76x10^6^ | 9.59x10^4^ | 9.02x10^4^ | 1.74x10^6^ | 7.78x10^4^ |
| Apr-17 | 6.53x10^7^ | 6.25x10^7^ | 6.51x10^7^ | 1.41x10^7^ | 1.76x10^6^ | 1.88x10^7^ | 5.09x10^6^ | 6.86x10^4^ | 5.20x10^4^ | 1.18x10^6^ | 1.33x10^4^ |
| May-17 | 1.21x10^9^ | 4.33x10^8^ | 4.35x10^8^ | 3.10x10^8^ | 2.15x10^6^ | 2.01x10^8^ | 2.85x10^6^ | 4.96x10^5^ | 4.80x10^5^ | 2.52x10^7^ | 2.96x10^3^ |
| Jun-17 | 1.29x10^8^ | 6.97x10^6^ | 8.05x10^7^ | 5.72x10^7^ | 5.40x10^5^ | 9.57x10^6^ | 5.63x10^5^ | 1.84x10^5^ | not tested | 3.66x10^6^ | 8.06x10^3^ |
| Jul-17 | 4.56x10^7^ | 6.19x10^7^ | 2.01x10^7^ | 5.39x10^7^ | 1.33x10^6^ | 5.39x10^6^ | 6.83x10^5^ | 1.89x10^5^ | 1.08x10^6^ | 2.68x10^6^ | 1.00x10^4^ |
| Aug-17 | 4.59x10^8^ | 6.25x10^5^ | 1.16x10^8^ | 7.92x10^7^ | 2.30x10^6^ | 3.55x10^6^ | 1.10x10^6^ | 1.10x10^5^ | negative | negative | 4.00x10^5^ |
| Mean | 2.28x10^8^ | 9.49x10^7^ | 8.92x10^7^ | 5.87x10^7^ | 3.02x10^6^ | 2.76x10^7^ | 1.12x10^7^ | 1.31x10^5^ | 3.20x10^5^ | 3.01x10^6^ | 5.33x10^4^ |
| SD | 3.26x10^8^ | 1.38x10^8^ | 1.17x10^8^ | 8.24x10^7^ | 3.10x10^6^ | 5.42x10^7^ | 1.70x10^7^ | 1.28x10^5^ | 4.10x10^5^ | 7.09x10^6^ | 1.07x10^5^ |

| Sampling date | Sediment (gc/g) | | | Shellfish (gc/g) | |
| --- | --- | --- | --- | --- | --- |
|  | Sed1 | Sed2 | Sed4 | SF1 | SF2 |
| Aug-16 | 3.54x10^3^ | 1.20x10^4^ | 2.33x10^3^ | 2.45x10^3^ | 1.16x10^4^ |
| Sep-16 | 2.17x10^3^ | 2.80x10^3^ | 3.07x10^3^ | 1.01x10^4^ | 1.36x10^4^ |
| Oct-16 | negative | 5.34x10^1^ | negative | negative | negative |
| Nov-16 | 1.91x10^4^ | 2.55x10^2^ | 1.91x10^4^ | not tested | 7.19x10^2^ |
| Dec-16 | 4.94x10^2^ | negative | 3.97x10^2^ | negative | not tested |
| Jan-17 | 5.30x10^2^ | 1.8103 | negative | 1.00x10^2^ | 9.54x10^2^ |
| Feb-17 | negative | 1.67x10^4^ | 2.12x10^2^ | 1.21x10^3^ | 1.80x10^3^ |
| Mar-17 | 5.35x10^2^ | 9.34x10^2^ | negative | 2.01x10^3^ | 8.40x10^3^ |
| Apr-17 | 1.77x10^3^ | negative | 7.23x10^2^ | 5.83x10^3^ | 9.79x10^3^ |
| May-17 | 2.12x10^3^ | 2.94x10^3^ | 1.06x10^3^ | 7.56x10^3^ | 4.88x10^3^ |
| Jun-17 | negative | negative | negative | negative | negative |
| Jul-17 | negative | 1.00x10^2^ | negative | 1.11x10^3^ | 5.04x10^2^ |
| Aug-17 | negative | negative | 5.55x10^2^ | 3.56x10^3^ | 2.48x10^3^ |
| Mean | 3.78x10^3^ | 4.18x10^3^ | 3.43x10^3^ | 3.77x10^3^ | 5.47x10^3^ |
| SD | 6.28x10^3^ | 5.98x10^3^ | 6.41x10^3^ | 3.36x10^3^ | 4.95x10^3^ |

Table S2. Spearman’s rank correlation coefficients (with p values in brackets) matrices for norovirus GI (NoV GI) and GII (NoV GII), sapovirus (SaV), adenovirus (AdV), JC polyomavirus (JCV) and crAssphage. *p<0.05; p<0.01; ***p<0.001.

**Wastewater influent (n = 49-52)**

| CrAss-  phage |  | 0.310*  (0.0306) | 0.494***  (3.92x10^-4^) | 0.263  (0.0683) | 0.340*  (0.0170) | 0.265  (0.0657) |
| --- | --- | --- | --- | --- | --- | --- |
| AdV | 0.310*  (0.0306) |  | 0.349*  (0.0114) | 0.204  (0.147) | 0.340*  (0.0140) | 0.549***  (2.92x10^-5^) |
| JCV | 0.494***  (3.92x10^-4^) | 0.349*  (0.0114) |  | 0.360***  (8.99x10^-4^) | 0.357**  (0.00967) | 0.314*  (0.0236) |
| NoVGI | 0.263  (0.0683) | 0.204  (0.147) | 0.360***  (8.99x10^-4^) |  | 0.0274  (0.846) | 0.321*  (0.0207) |
| NoVGII | 0.340*  (0.0170) | 0.340*  (0.0140) | 0.357**  (0.00967) | 0.0274  (0.846) |  | 0.295*  (0.0339) |
| SaV | 0.265  (0.0657) | 0.549***  (2.92x10^-5^) | 0.314*  (0.0236) | 0.321*  (0.0207) | 0.295*  (0.0339) |  |
|  | CrAss-  phage | AdV | JCV | NoVGI | NoVGII | SaV |

**Wastewater effluent (n = 37-39)**

| CrAss-  phage |  | 0.128  (0.446) | 0.674***  (2.43x10^-7^) | 0.202  (0.229) | 0.429**  (0.00825) | 0.211  (0.208) |
| --- | --- | --- | --- | --- | --- | --- |
| AdV | 0.128  (0.446) |  | 0.171  (0.296) | 0.0796  (0.628) | -0.0126  (0.939) | 0.232  (0.155) |
| JCV | 0.674***  (2.43x10^-7^ | 0.171  (0.296) |  | 0.0897  (0.585) | 0.543***  (3.91x10^-4^) | 0.217  (0.184) |
| NoVGI | 0.202  (0.229) | 0.0796  (0.628) | 0.0897  (0.585) |  | 0.0931  (0.571) | 0.0275  (0.867) |
| NoVGII | 0.429**  (0.00825) | -0.0126  (0.939) | 0.543***  (3.91x10^-4^) | 0.0931  (0.571) |  | 0.232  (0.154) |
| SaV | 0.211  (0.208) | 0.232  (0.155) | 0.217  (0.184) | 0.0275  (0.867) | 0.232  (0.154) |  |
|  | CrAss-  phage | AdV | JCV | NoVGI | NoVGII | SaV |

**Surface water (n = 51-52)**

| CrAss-phage |  | -0.0210  (0.883) | 0.489***  (3.03x10^-4^) | 0.0845  (0.554) | 0.0670  (0.639) | -0.0686  (0.631) |
| --- | --- | --- | --- | --- | --- | --- |
| AdV | -0.0210  (0.883) |  | 0.418**  (0.00216) | 0.237  (0.0912) | -0.00166  (0.991) | 0.0801  (0.571) |
| JCV | 0.489***  (3.03x10^-4^) | 0.418**  (0.00216) |  | 0.0653  (0.644) | 0.145  (0.303) | -0.0240  (0.865) |
| NoVGI | 0.0845  (0.554) | 0.237  (0.0912) | 0.0653  (0.644) |  | 0.253  (0.0704) | -0.0651  (0.645) |
| NoVGII | 0.0670  (0.639) | -0.00166  (0.991) | 0.145  (0.303) | 0.253  (0.0704) |  | 0.202  (0.149) |
| SaV | -0.0686  (0.631) | 0.0801  (0.571) | -0.0240  (0.865) | -0.0651  (0.645) | 0.202  (0.149) |  |
|  | CrAss-  phage | AdV | JCV | NoVGI | NoVGII | SaV |

**Sediment (n = 38-39)**

| CrAss-phage |  | 0.369*  (0.0229) | 0.0861  (0.605) | | -0.0801  (0.630) | | 0.242  (0.143) | |  |
| --- | --- | --- | --- | --- | --- | --- | --- | --- | --- |
| AdV | 0.369*  (0.0229) |  | 0.418**  (0.00841) | | 0.317*  (0.0491) | | -0.128  (0.436) | |  |
| JCV | 0.0861  (0.605) | 0.418**  (0.00841) |  | | 0.351*  (0.0287) | | 0.238  (0.144) | |  |
| NoVGI | -0.0801  (0.630) | 0.317*  (0.0491) | 0.351*  (0.0287) | |  | | -0.134  (0.413) | |  |
| NoVGII | 0.242  (0.143) | -0.128  (0.436) | 0.238  (0.144) | | -0.134  (0.413) | |  | |  |
|  | CrAss-  phage | AdV | | JCV | | NoVGI | | NoVGII | |

**Shellfish (blue mussel)**

| CrAss-phage |  | 0.374  (0.0703) | | -0.354  (0.0886) | | -0.0787  (0.709) | -0.129  (0.542) |
| --- | --- | --- | --- | --- | --- | --- | --- |
| AdV | 0.374  (0.0703) |  | | 0.193  (0.342) | | 0.459*  (0.0187) | 0.143  (0.481) |
| JCV | -0.354  (0.0886) | 0.193  (0.342) | |  | | 0.253  (0.209) | 0.125  (0.537) |
| NoVGI | -0.0787  (0.709) | 0.459*  (0.0187) | | 0.253  (0.209) | |  | 0.0119  (0.952) |
| NoVGII | -0.129  (0.542) | 0.143  (0.481) | | 0.125  (0.537) | | 0.0119  (0.952) |  |
|  | CrAss-  phage | AdV | JCV | | NoVGI | | NoVGII |

Figure S1. Linear regression between JCV and crAssphage in different wastewater (WW) influent, effluent and surface water (SW) samples. Lines represent linear regression analysis with the R^2^ values presents at the bottom of each panel.
